# Effect of Perceived Self-Vulnerability on Prostate Cancer Screening Uptake and Associated Factors: A Cross-Sectional Study of Public Health Facilities in Western Kenya

**DOI:** 10.5334/aogh.3064

**Published:** 2022-02-17

**Authors:** Charles Oduor Opondo, Patrick Ogola Onyango, Collins Otieno Asweto

**Affiliations:** 1 Department of Public Health, Maseno University, Private Bag, Maseno, KE; 2 School of Physical and Biological Sciences, Maseno University, Private Bag, Maseno, KE; 3 Department of Community Health, School of Nursing, University of Embu, P. O. Box 6 – 60100, Embu, KE

## Abstract

**Background:**

Perceived self-vulnerability to prostate cancer is known to influence screening uptake among men in the general population. However, knowledge gap persists on the influence of perceived self-vulnerability to prostate cancer on uptake of screening among male health workers; a demographic that has health insurance and is assumed to have knowledge of screening services for prostate cancer.

**Objective:**

This study aimed to assess the effect of perceived self-vulnerability to prostate cancer on screening uptake among male health workers in Kisumu County, western Kenya.

**Methods:**

This was a hospital-based cross-sectional study with a descriptive and analytical design. A modified self-administered questionnaire on self-vulnerability was issued to 197 male health workers who were randomly sampled from a study population of 336 eligible participants. The study was conducted at purposively selected public health facilities.

**Findings:**

Level of self-reported screening uptake was 27%. Rural residence (AOR = 0.71: 95% CI, 0.32–1.57, p = 0.019), education level (AOR = 5.01; 95% CI, 1.2–20.86, p = 0.027), participant’s lack of knowledge about screening services covered by health insurance schemes of which they are members (AOR = 0.2, 95% CI, 0.08–0.5, p = 0.001), good perception of health status (AOR = 4; 95% CI: 1.52–10.53, p = 005) were determinants of screening uptake for prostate cancer. Perceived self-vulnerability to prostate cancer didn’t influence screening uptake of participants (p < 0.05). Participants from rural set-up had a higher likelihood of perceiving themselves to be at risk of prostate cancer (AOR = 2.35, 95% CI, 1.17–4.72, p < 0.05) compared to those form urban settings. Old age of 60 years and above (AOR = 3.5, 95% CI: 0.3–40.98, p < 0.002) was predictive of perceived self-vulnerability.

**Conclusion:**

Findings from this study showed low uptake of screening and low perceived self-vulnerability to prostate cancer. Perceived self-vulnerability did not influence screening uptake for prostate cancer. Screening knowledge of prostate cancer as covered by health insurance, good perception of health status and level of education should be integrated in screening programs that are individualized on the basis of personal preferences and informed decision making regarding the uncertainty of benefit and the associated harms of screening.

## Introduction

Prostate cancer is the most frequently diagnosed adenocarcinoma and the second cause of cancer deaths after lung cancer among men globally [1]. According to [2], an estimated 1.1 million cases and 307 500 deaths occurred in the year 2012. Prostate cancer incidence rates in developed countries are higher than rates in developing countries [3]. However, mortality rates due to the disease in developed countries are lower than those in developing countries. This variation is attributed to widespread uptake of screening services in developed countries, late stage diagnosis in developing countries and differences in male life expectancies across countries [1].

According to Rebbeck and Colleagues [4], mortality rates from prostate cancer is normally higher in black African populations. Similar differences were observed in the distribution patterns between regions in northern and sub-Saharan Africa [5]. For instance, previous statistics indicate prostate cancer incidence and mortality rates in north Africa as 10.6 and 7.0 per 100 000 respectively [1]. In comparison, those in Sub-Saharan Africa were 34.3 and 22.1 per 100 000 respectively. This implies a higher burden of the disease in Sub-Saharan Africa. In Kenya, for example, age standardized mortality rate for prostate cancer is 31.6 per 100 000 men [1].

Prostate cancer screening is an attempt to presumptively identify the disease in asymptomatic individuals in the population. The three methods of screening are the measurement of prostate specific antigen in serum, digital rectal examination and transurethral ultrasonography. Digital Rectal Examination (DRE) has low sensitivity when used alone hence it should be applied together with Prostate Specific Antigen (PSA) method to improve detection rate [5]. However, a final diagnosis of prostate cancer can only be confirmed through a biopsy and subsequent microscopy [6]. Early diagnosis allows for more effective treatment, increases survival rate thereby reducing cost of care and risk of death [7,8]. It is believed that more than 69% of prostate cancer deaths can be prevented during the first five years following diagnosis [8]. Conflicting results from the two large randomized clinical trials; the European Randomized Study of Screening for Prostate Cancer [ERSPC] showed 20% reduction of mortality among men in the screening group compared to those in the control group [9], while the randomized prospective trial (Prostate, Lung, Colorectal, Ovarian Trial [PLCO]) showed no benefit among men in the screening group compared to those in the control group [8]. Consequently, the benefits of prostate cancer screening using PSA and DRE is still controversial within the medical and scientific community due to the fact that each test is more likely to detect cancers of unknown clinical significance and fail to distinguish life threatening tumors which might remain in a latent stage, making it difficult to understand the natural progression of the disease. Thus, death rates due to causes other than prostate cancer may become clinically evident among men due to slow growth of prostate tumors [10,11]. In addition, treatment can result to complications and undesirable side effects including bowel injury, impotence and incontinence [11,12]. Despite these controversies, the American Cancer Society emphasizes informed decision making at age 45 years for high risk men for example, African Americans and those with family history and 40 years of age for those men with extensive family history [6], while the Kenya National Screening guidelines recommends against mass screening but rather a highly individualized screening only after the benefits and limitations of screening have been explained for an informed decision making among men aged 40 years and above [13].

In spite of high mortality rate due to prostate cancer in Sub Saharan Africa, there is no centralized, systematic, population-based prostate cancer screening program for early detection in many African countries including Kenya [14,15,16,17]. For example, in Kenya, majority (87.5%) of patients attend hospitals when prostate cancer disease has reached an advanced stage [18]. A study at Kenyatta National Teaching and Referral Hospital showed that only 23.7% of male patients aged 40 years and above self-reported to have undergone prostate cancer screening [19]. However, another household survey in Nairobi found that only 4.1% of respondents reported to have ever been screened for prostate cancer [20]. These figures are lower than those reported in men of similar age cohort in other countries such as United States of America and European countries [8,9].

Previous studies have reported poor uptake of prostate cancer screening among men in the general population [14,20,21,22,23,24,25]. In addition to knowledge and economic constraints, an individual’s perception of health risk is an equally important predictor of uptake of cancer screening [15,26,27,28]. Previous studies have revealed that a number of socio-demographic factors such as age, marital status, family history, health insurance, and knowledge predict perception of self-vulnerability among men at risk of the disease in the general population [14,15,16,29,30,31]. Moreover, perception of self-vulnerability has been shown to influence cancer prevention in the general population [15,21,27,28]. However, the influence of these factors and perceived self-vulnerability to prostate cancer on uptake of screening among male healthcare workers is unknown. The main objective of the present study was to determine uptake of prostate cancer screening and perceived self-vulnerability among male health workers in public health facilities in Kisumu County, Kenya. Specifically, we determined the level of self-reported uptake of screening for prostate cancer, socio-demographic characteristics associated with uptake of prostate cancer screening, examined the influence of perceived self-vulnerability to prostate cancer on screening uptake, and identified socio-demographic predictors of perceived self-vulnerability to prostate cancer among male health workers.

## Material and Methods

### Participants and settings

The study population consisted of male healthcare workers aged 40 years and above distributed across public health facilities in Kisumu County, western Kenya. We excluded male health workers who were absent from their work stations during the study period. The sample size was established using sample size calculation formula by Fisher et al [32] for a finite population of 336 eligible male health care workers, where z² = the standard normal deviate (1.96) at 95% confidence interval, n = the desired sample size, p = proportion in the target population with characteristic of interest set at 0.5, q = 1–p, d² = level of statistical significance set at 0.05. Since the target population in this study was less than 10 000, the formula to find nf = desired sample size when the population is less than 10 000 was used to calculate a sample size of 179. A standard 10% non-response was added to obtain a final sample of 197 participants who were selected and included in the study using random sampling technique. Purposive sampling was used to select health facilities with the highest number of eligible study participants.

### Survey instrument

We adopted a cross-sectional study design using a modified self-administered questionnaire on Perceived Vulnerability to Disease (PVD) scale [33]. The original scale was designed to assess individual differences in chronic concerns about the transmission of diseases among 1560 undergraduate students from United States of America. In this study, perceived self-vulnerability questionnaire on a 5 point rating scale response (1 = strongly disagree to 5 = strongly agree) was modified to measure perceived self-vulnerability to prostate cancer. Prior to the actual survey, the questionnaire was pre-tested at selected health facilities in the neighboring County of Kakamega. The raw data was entered in STATA version 14 and subjected to a reliability scale analysis that revealed a Cronbach’s alpha of 0.67 which was approximately 0.70 considered as an acceptable gold standard. Therefore, participants were asked to rate their level of agreement. Rating across the 15 items were averaged to create a composite PVD score with higher scores indicating greater PVD. Data was collected in the period between November 2018 and January 2019.

### Data Analysis

Data was entered in a password-protected computer and verified for completeness. The complete data was then exported to STATA version 14.0 where both descriptive and inferential statistical analyses were performed. Chi-square test was used to examine the association between perceived self-vulnerability and screening uptake while univariate and logistic regression analysis was used to identify the socio-demographic predictors of screening uptake and perceived self-vulnerability to prostate cancer. *P*-value ≤ 0.05 was considered statistically significant.

### Ethical considerations

Ethical clearance was obtained from Maseno University Ethics Review Committee (REF No.MSU/DRPI/MUERC/00585/18) and Jaramogi Oginga Odinga Teaching and Referral Hospital Ethics Review Committees (REF No.ERC.IB/VOL.1/616). Permission to conduct the study was obtained from the Director of health of Kisumu County. Prior to the study, informed consent was sought from participants. They were provided with full information and it was confirmed that they could comprehend the questions. Confidentiality was ensured through anonymity using unique numbers, privacy was maintained during interviews and voluntary participation was allowed.

## Results

### Socio-demographic characteristics

***Table 1*** presents data on socio-demographic characteristics of the respondents. The youngest health care provider interviewed was 40 years old and the oldest was 71 years old. The median age was 48 years (IQR = 9). Sixty-six percent of those interviewed were in the 40 - 50 year age group, 30% were in the 51–60 year age group and 4% were aged 60 years and above. In terms of level of education, 21% had certificate, 56% diploma, 17% bachelor degree and 6% masters’ degree as the highest level of education. In terms of residence, 56% lived in urban areas while 44% in rural areas. More than two-thirds (191; 97%) were married; and by religion, nearly all the respondents were Christians (98%).

**Table 1:** Socio demographic characteristics of male health workers in Kisumu County (n = 197).


SOCIO DEMOGRAPHIC CHARACTERISTICS OF THE RESPONDENTS:	RESPONDENTS, N	PROPORTION (%)

**Age (in years)**		

40–50	130	66

51–60	59	30

Above 60	8	4

**Level of Education**		

Certificate	41	21

Diploma	110	56

Graduate	34	17

Postgraduate	12	6

**Residence**:		

Urban	111	56

Rural	86	44

**Marital Status**:		

Unmarried	3	1.5

Married	191	97

Separated	3	1.5

**Religion**:		

Muslim	4	2

Christian	193	98


### Level of self-reported prostate uptake of cancer screening

In determining the uptake level of screening for prostate cancer, only 27% of respondents self-reported to have undergone screening for prostate cancer while 73% were not screened.

### Socio demographic characteristics associated with uptake of prostate cancer screening

In assessing the association between socio demographic factors and uptake of screening services, a univariate analysis and multivariate logistic regression analysis of independent variables and screening uptake was performed and presented in ***Table 2***. Having a diploma level of education was associated with 5.01 times (AOR = 5.01; 95% CI; 1.20–20.86) higher odds of self-reported uptake of screening services compared to those with certificate level of education. Participants who perceived their health status as good were 4 times (AOR = 4; 95% CI: 1.52, 10.53) more likely to report as having undergone screening services than those with fair perception of health status. Respondents who were not aware that their health insurance covered cost of screening were 0.2 (AOR = 0.2; 95% CI: 0.08–0.5) times less likely to have gone for screening services than those with knowledge that health insurance emancipated them from cost of screening. Participants from rural setting were less likely to self- report having undergone screening when compared with their urban counterparts (AOR = 0.71: 95CI, 0.32–1.57, p = 0.019)

**Table 2:** Socio-demographic factors associated with uptake of prostate cancer screening by study respondents (n = 197)


VARIABLES	TOTAL	NOT SCREENED	SCREENED	UNIVARIABLE OR (95% CI)	*P-VALUE*	MULTIVARIABLE AOR (95% CI)	*P-VALUE*

**Age Category**							

40 to 50	130	96 (73.85)	34 (26.15)	Ref			

51 to 60	59	42 (71.19)	17 (28.81)	1.14 (0.58–2.27)	0.703		

Above 60	8	5 (62.5)	3 (37.5)	1.69 (0.38–7.47)	0.486		

**Education Level**							

Certificate	41	35 (85.37)	6 (14.63)	Ref		Ref	

Diploma	110	75 (68.18)	35 (31.82)	3.97 (1.31–12.05)	**0.015**	5.01 (1.2–20.86)	**0.027**

Graduate	34	25 (73.53)	9 (26.47)	3.06 (0.85–11.07)	0.088	1.83 (0.4–8.37)	0.433

Postgraduate	12	8 (66.67)	4 (33.33)	4.25 (0.87–20.75)	0.074	2.23 (0.35–14.02)	0.393

**Residence**							

Urban	111	74 (66.67)	37 (33.33)	Ref		Ref	

Rural	86	69 (80.23)	17 (19.77)	0.49 (0.25–0.95)	**0.036**	0.71 (0.32–1.57)	**0.019**

**Marital Status**							

Unmarried	3	2 (66.67)	1 (33.33)	Ref			

Married	191	139 (72.77)	52 (27.23)	0.75 (0.07–8.43)	0.814		

Separated	3	2 (66.67)	1 (33.33)	1 (0.03–29.81)	1.000		

**Religion**							

Muslim	4	2 (50)	2 (50)	Ref			

Christian	193	141 (73.06)	52 (26.94)	0.37 (0.05–2.69)	0.325		

**Family history for prostate Cancer**							

Yes	27	15 (65.22)	8 (34.78)	Ref			

No	123	88 (71.54)	35 (28.46)	0.75 (0.29–1.92)	0.542		

Don’t know	51	40 (78.43)	11 (21.57)	0.52 (0.17–1.53)	0.232		

**Perception of health status**							

Fair	53	45 (84.91)	8 (15.09)	Ref		Ref	

Good	132	90 (68.18)	42 (31.82)	2.62 (1.14–6.06)	**0.024**	4 (1.52–10.53)	**0.005**

Excellent	12	8 (66.67)	4 (33.33)	2.81 (0.68–11.59)	0.152	3.93 (0.81–19.17)	0.091

**Health Insurance Ownership**							

Yes	186	135 (72.58)	51 (27.42)	Ref			

No	11	8 (72.73)	3 (27.27)	0.99 (0.25–3.89)	0.992		

**Respondent’s knowledge that health insurance covers cost of screening**							

Yes	104	65 (62.5)	39 (37.5)	Ref		Ref	

No	13 13	9 (69.23)	4 (30.77)	0.74 (0.21–2.57)	0.636	0.88 (0.23–3.39)	0.856

Don’t know	69	61 (88.41)	8 (11.59)	0.22 (0.09–0.5)	**<0.0001**	0.2 (0.08–0.5)	**0.001**


### Influence of perceived self-vulnerability to prostate cancer on screening uptake

Analysis of a 5 – point Likert scale for perception of self-vulnerability revealed that only 15% of the health care workers interviewed agreed that they were vulnerable to prostate cancer during the next 5 years (***Table 3***). Sixty-five percent agreed that they could get prostate cancer if they did not take any preventive measures such as regular screening while 44% agreed that they were worried about the possibility of getting the disease. About 9% of male health care workers reported that they come from families with a history of prostate cancer disease whereas 23% agreed that they were afraid they might be diagnosed with prostate cancer. Collectively, findings revealed that 55% of the health care workers perceived themselves as less vulnerable while 45% of participants perceived themselves as vulnerable to prostate cancer disease (***Table 4***).

**Table 3:** Level of agreement and disagreement to perceived self-vulnerability of respondents (n = 197) to prostate cancer.


PERCEIVED SELF-VULNERABILITY	STRONGLY DISAGREE	DISAGREE	DON’T KNOW	AGREE	STRONGLY AGREE

There is a good chance I will get prostate cancer during the next 5 years	38 (19%)	50 (25%)	80 (41%)	22 (11%)	7 (4%)

I think I can get prostate cancer if I don’t take any preventive measure such as regular screening	26 (13%)	23 (12%)	20 (10%)	98 (50%)	30 (15%)

I worry about the possibility of getting prostate cancer disease	19 (10%)	69 (35%)	22 (11%)	63 (32%)	24 (12%)

I have a history of susceptibility to prostate cancer	48 (24%)	83 (42%)	50 (25%)	11 (6%)	5 (3%)

I am afraid I might be diagnosed with prostate cancer disease	40 (20%)	69 (35%)	44 (22%)	35 (18%)	9 (5%)


**Table 4:** Association between perceived self-vulnerability to prostate cancer and uptake of screening uptake among respondents (n = 197)


VARIABLE	N (%)	NOT SCREENED N (%)	SCREENED N (%)	CHI- *P-VALUE*	COR (95% CI)	*P-VALUE*

**Perception**						

Low Perception	109 (55.33)	83 (76.15)	26 (23.85)	0.213	Ref	0.214

High Perception	88 (44.67)	60 (68.18)	28 (31.82)		1.49 (0.79–2.79)	


One Shapiro-Wilk test revealed that perceived self-vulnerability scores were not distributed normally. We used Pearson’s chi-square test of independence to test for association between perception of self-vulnerability to prostate cancer and uptake of screening. Results revealed that perceived self-vulnerability to prostate cancer had no effect on uptake of screening by study participants (*χ*² (1) = 0.2140: COR = 1.49; 95 CI; 0.79–2.79, *p* > 0.05). This is displayed on ***Table 4***.

### Socio-demographic predictors of perceived self-vulnerability to prostate cancer

Findings from a univariate analysis revealed that old age (above 60 years) (COR = 10.17:95CI, 1.22–85.09, p = 0.032), diploma education (COR = 0.35:95CI, 0.16–0.74, p = 0.007) rural residence (COR = 2.44:95CI, 1.37–4.36, p = 0.002) and respondent’s lack of knowledge about screening service covered by health insurance (COR = 2.32:95CI, 1.24–4.32, p = 0.008) were factors independently associated with perceived self-vulnerability of respondents to prostate cancer. When these independent variables were further subjected to a multivariate logistic regression analysis, old age (AOR = 3.5: 95CI, 0.3–40.98, p = 0.002) and rural residence (AOR = 2.35:95CI, 1.17–4.72, p = 0.016) were highly predictive of perceived self-vulnerability to prostate cancer disease as displayed on ***Table 5***.

**Table 5:** Predictor variables of perceived self-vulnerability of respondents to prostate cancer (n = 197).


PREDICTOR VARIABLES	N	LOW PERCEPTION N (%)	HIGH PERCEPTION N (%)	UNIVARIABLE COR (95% CI)	*P-VALUE*	MULTIVARIABLE AOR (95% CI)	*P-VALUE*

**Age Category**							

40 to 50	130	77 (59.23)	53 (40.77)	Ref		Ref	

51 to 60	59	31 (52.54)	28 (47.46)	1.31 (0.71–2.44)	0.390	1.19 (0.59–2.42)	0.623

Above 60	8	1 (12.5)	7 (87.5)	10.17 (1.22–85.09)	**0.032**	3.5 (0.3–40.98)	**0.002**

**Education Level**							

Certificate	41	14 (34.14)	27 (64.85)	Ref		Ref	

Diploma	110	69 (62.73)	41 (37.27)	0.35 (0.16–0.74)	**0.007**	0.75 (0.3–1.85)	0.535

Graduate	34	20 (58.82)	14 (41.18)	0.41 (0.16–1.05)	0.064	0.94 (0.32–2.79)	0.918

Postgraduate	12	6 (50.0)	6 (50.0)	0.58 (0.16–2.16)	0.420	1.73 (0.4–7.54)	0.466

**Residence**							

Urban	111	72 (64.86)	39 (35.14)	Ref		Ref	

Rural	86	37 (43.02)	49 (56.98)	2.44 (1.37–4.36)	**0.002**	2.35 (1.17–4.72)	**0.016**

**Marital Status**							

Unmarried	3	2 (66.67)	1 (33.33)	Ref			

Married	191	107 (56.02)	84 (43.98)	1.57 (0.14–17.61)	0.715		

Separated	3	0 (0)	3 (100)	1			

**Religion**							

Muslim	4	3 (75)	1 (25)	Ref			

Christian	193	106 (54.92)	87 (45.08)	2.46 (0.25–24.09)	0.439		

**Family history of prostate cancer**							

Yes	23	13 (56.52)	10 (43.48)	2.03 (0.73–5.69)	0.176	2.15 (0.7–6.62)	0.183

No	123	59 (47.97)	64 (52.03)	2.87 (1.41–5.83)	**0.004**	1.87 (0.82–4.28)	0.139

Don’t know	51	37 (72.55)	14 (27.45)	Ref		Ref	

**Perception of health status**							

Fair	53	25 (47.17)	28 (52.83)	Ref			

Good	132	76 (57.58)	56 (42.42)	0.66 (0.35–1.25)	0.200		

Excellent	12	8 (66.67)	4 (33.33)	0.45 (0.12–1.66)	0.230		

**Health Insurance Ownership**							

Yes	186	107 (57.53)	79 (42.47)	Ref			

No	11	2 (18.18)	9 (81.82)	0.16 (0.03–0.78)	**0.023**		

**Respondents’ Knowledge that health insurance covers cost of screening**							

Yes	104	68 (65.38)	36 (34.62)	Ref		Ref	

No	13	8 (61.54)	5 (38.46)	1.18 (0.36–3.87)	0.784	0.78 (0.22–2.83)	0.706

Don’t Know	69	31 (44.93)	38 (55.07)	2.32 (1.24–4.32)	**0.008**	1.55 (0.77–3.1)	0.219


## Discussion

Prostate cancer screening for early detection and prompt treatment remains controversial within the medical and scientific community. Nonetheless, this study investigated uptake of prostate cancer screening and perceived self-vulnerability of male health care workers at public health facilities in Kisumu County, Kenya.

Out of a total of 197 male health care workers, only 27% reported to have undergone screening for prostate cancer by PSA. This implies that male health care workers do not go for annual screening in Kenya. These results are consistent with a previous study among Nigerian doctors which found an uptake level of 1.4% for prostate cancer screening [34]. The Nigerian rate is lower than 27% uptake level found in this study. Differences in the level of uptake of prostate cancer screening between the current study and that by [34] may be explained by the fact that the current study focused on male health care workers from different health service cadres unlike the previous study which focused only on male doctors for the prostate component of their study.

In this study, uptake of screening was not associated with age, religion, and marital status among study respondents. Concerning age, the lack of association is contrary to findings of two American studies which found that old age had a significant positive effect on screening for prostate cancer [26,35]. The conflicting results on age may be explained by a function of age distribution in this study versus a previous study [35] where participants aged 50 years and above constituted 75% of the sample compared with 39% of the sample in the current study.

As for education, it was surprising to see that participants with diploma level of education were more likely to self-report uptake of screening services than those with graduate and postgraduate education level. This is in contrast to what is commonly reported in extant literature where those with higher education levels are more likely to report screening in a positive light compared to those with lower education level [26,36]. The contradictory findings in the current study might be because a majority of participants in the sample were those with diploma level of education compared to previous literature findings. In this study, good perception of health **s**tatus was significantly associated with screening uptake for prostate cancer. Compared to those respondents with good perception of health status, those in the excellent perception of health status category were less likely to undergo screening. These findings compare with those from a previous study which reported that fair perception of health status was significantly and positively associated with intention to undergo screening for prostate cancer [26]. It could be argued that absence of health problems on male prostate gland may act as a barrier to undergo screening among participants in the excellent health status category while presence of health problems can act as cues to action for screening. Place of residence was significantly associated with uptake of prostate cancer screening by male healthcare workers. Majority of participants from rural settings self-reported as less likely to have screened for prostate cancer compared to those from urban settings. Previous studies have found a wide geographical variation in terms of screening with urban residents more likely to report screening than those from rural areas [29,37]. This disparity in screening uptake between urban versus rural residents may be explained by lack of availability and accessibility of screening services in rural and remote areas. Respondents who did not know that their health insurance cover emancipated them from cost of screening services were less likely to undergo screening in the current study. Studies elsewhere [26,29] have reported health insurance as a predictor of access to screening services which is in contrast to the current study. The conflicting results may be explained by participants’ knowledge and fear of discomfort, harms and benefits of screening as well as complications of subsequent treatment for prostate cancer despite ownership of health insurance for screening.

In this study, majority of respondents perceived themselves as not vulnerable to prostate cancer disease. This compares with findings of previous study [38] which showed that medical workers gave a lower risk perception rating than non-medical workers for various types of diseases including cancer.

With regard to the perception of self-vulnerability and its role in predicting uptake of prostate cancer screening among participants, findings of this study did not reveal any association between perception of self-vulnerability and uptake of screening services. This may be explained that male health care workers perceived themselves as vulnerable to the harms and discomfort from screening as opposed to being vulnerable to prostate cancer disease. In addition, male health care workers may not view prostate cancer as a serious health condition but rather a problem of the clients they serve. This phenomenon is referred to as optimistic bias. Optimistic bias occurs when individuals believe that they are less at risk to an adverse health event compared to others. They are thus convinced that a disease can only affect others but not them. Knowledge of the discomfort, risks and harms associated with screening for prostate cancer as well as optimistic bias among male health workers may thus explain why previous studies have reported an association between perceived self-vulnerability and prostate cancer screening in the general population [20,21,27,28,39,40] but not in the present study.

Male health care workers aged 60 years and above were more likely to perceive themselves as being at risk of prostate cancer compared to their younger counterparts. This result suggests a disparity in perception of self-vulnerability in favor of older men. The disparity in age evident in perceived self-vulnerability to prostate cancer may be attributed to experiential construct of risk perception. In other words, healthcare workers aged 60 years and above are more likely to have dealt with more cases of prostate cancer to the extent that they worry about it. These findings are comparable with those from a study elsewhere which established that older black males were more likely to perceive themselves as being vulnerable to cancer diagnoses [41]. An interesting finding from the current study is that participants from rural settings were more likely to perceive themselves as more vulnerable to prostate cancer than those from urban settings. This is the first study to report such a finding hence it has implications for further qualitative research.

## Conclusion

Male health workers uptake of screening for prostate cancer remains low despite having health insurance cover that exempts them from the cost of screening using PSA. There is a disparity in screening uptake between rural and urban dwellers. Male health care workers aged 60 years and above viewed themselves as being more at risk of prostate cancer than their younger colleagues. Perceived self-vulnerability to prostate cancer was not associated with uptake of screening services. Therefore, screening programs for prostate cancer should individualized on the basis of personal preferences and informed decision making regarding the uncertainty of benefit and the associated harms of screening. Further research is needed to understand perceived self-vulnerability to prostate cancer in relation to harms and benefits of screening for an informed decision making in this demographic at risk of disease.

## Data Accessibility Statement

Raw data used to support findings of this study are available upon request from the corresponding author.

## Additonal File

The additonal file for this article can be found as follows:

10.5334/aogh.3064.s1 Effect of Perceived Self-Vulnerability on Prostate Cancer Screening Uptake and Associated Factors: A Cross-Sectional Study of Public Health Facilities in Western Kenya. The additional file has data that represent socio demographic variables for prostate cancer screening uptake and perceived self - vulnerability to prostate cancer among male health workers aged 40 years and above who were recruited from public health facilities in western Kenya.
